# Antibiotic resistance in conflict settings: lessons learned in the Middle East

**DOI:** 10.1093/jacamr/dlz002

**Published:** 2019-04-10

**Authors:** Rupa Kanapathipillai, Nada Malou, Joost Hopman, Conor Bowman, Nagwan Yousef, Justine Michel, Nagham Hussein, Patrick Herard, Janet Ousley, Clair Mills, Caroline Seguin, Malika Saim

**Affiliations:** 1 Médecins Sans Frontières, Operational Center Paris, Paris, France; 2 Department of Medical Microbiology and Radboud Center for Infectious Diseases, Radboud University Medical Center, Nijmegen, The Netherlands; 3 Médecins Sans Frontières, Operational Center Amsterdam, Amsterdam, The Netherlands; 4 Médecins Sans Frontières, Amman, Jordan; 5 Médecins Sans Frontières, Aden, Yemen

## Introduction

Médecins Sans Frontières (MSF) has designed context-adapted antibiotic resistance (ABR) responses in countries across the Middle East. There, some health systems have been severely damaged by conflict resulting in delayed access to care, crowded facilities and supply shortages. Microbiological surveillance data are rarely available, but when MSF laboratories are installed we often find MDR bacteria at alarming levels.^1^ In MSF’s regional hospital in Jordan, where surgical patients have often had multiple surgeries in field hospitals before reaching definitive care (often four or more), MSF microbiological data analysis reveals that, among Enterobacteriaceae isolates, third-generation cephalosporin and carbapenem resistance is 86.2% and 4.3%, respectively; MRSA prevalence among *Staphylococcus aureus* is 60.5%; and resistance types and rates are similar in patients originating from Yemen, Syria and Iraq.^1–3^ These trends compel MSF to aggressively prevent and diagnose ABR in Jordan, providing ABR lessons that inform the antibiotic choices, microbiological diagnostics and anti-ABR strategies in other Middle Eastern MSF trauma projects (such as Yemen and Gaza).

As a result, MSF has created a multifaceted, context-adapted, field experience-based, approach to ABR in hospitals in Middle Eastern conflict settings. We focus on three pillars: (1) infection prevention and control (IPC); (2) microbiology and surveillance; and (3) antibiotic stewardship.

### Pillar 1: infection prevention and control

**Figure 1.**
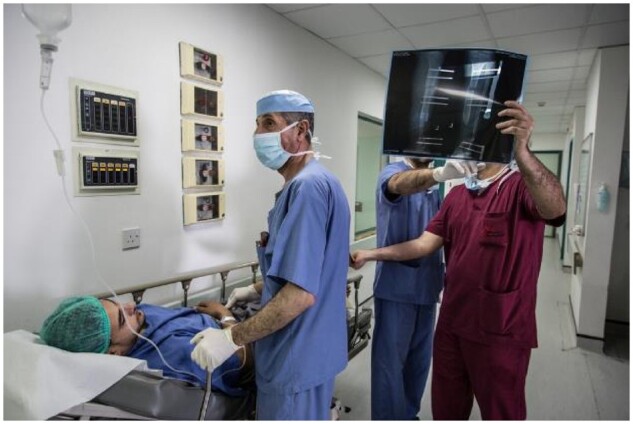
In MSF’s hospital in Amman, Jordan, a Syrian patient is prepared for surgery on his leg after being shot. Conflict-affected patients at this facility come from many places (Iraq, Yemen, Syria and Gaza), usually having had multiple other surgeries before reaching MSF, and are often infected with MDR bacteria. © Chris Huby/MSF.

We see many nosocomial MDR infections related to poor IPC.^4^^,^^5^ Since changing IPC-related behaviours can be extremely challenging, MSF’s approach standardizes and simplifies IPC measures to focus on high-impact interventions tailored to low-resource settings. IPC innovations (for example, a hand hygiene phone application and online stepwise IPC improvement tool) make MSF IPC programmes easier to implement and more sustainable. We focus heavily on three critical IPC areas for frontline health workers: hand hygiene (including appropriate glove use), cleaning and disinfecting surfaces and medical devices and transmission-based precautions. Our hospital-built environments are improved by arranging beds adequately, creating areas for cohorting MDR patients and securing critical resources (clean water, alcohol-based hand sanitizers, reliable cleaning supplies for decontamination and waste disposal and improved sterilization services)(Figure 1). Ongoing collaboration with Ministries of Health remains essential to improving long-term sustainability. Training and education are integrated into multimodal interventions (infrastructure improvements, audits, monitoring and feedback and adequate communication). MSF also provides sterilization autoclaves to partner non-governmental organizations, field-level training and technical support for quality control. We will next focus on strengthening health systems, improving surveillance and MDR outbreak management.

### Pillar 2: microbiology and surveillance

The second pillar of our approach improves access to microbiological diagnostic tools. MSF has established reliable microbiology laboratories in Amman (Jordan), Koutiala (Mali) and Aden (Yemen) with simplified protocols and quality control measures in place. All of these sites have formidable challenges: staff with microbiological expertise (especially for conducting antibiotic susceptibility testing using phenotypic methods) are frequently lacking. Technology can be prohibitively expensive, require reagents with limited shelf-lives and be ill-adapted to resource-limited settings without regular electricity supplies or a cold chain for sensitive items (such as antibiotic discs).^6^ Procuring quality-assured products is likewise an obstacle, often resulting in supply shortages when relying on imported shipments.

**Figure 2.**
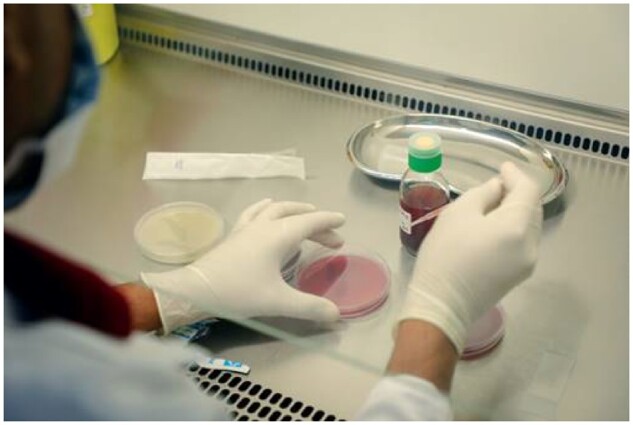
War-wounded surgical patients at MSF’s facility in Aden, Yemen who are found to be infected with drug-resistant bacteria are transferred to an isolation ward to prevent further hospital-based transmission. © Ehab Zawati/MSF.

Microbiological capacity, including human resources, expertise, and coordination, must be expanded in resource-limited and complex settings. Innovations such as MSF’s Mini-lab, which simplifies microbiological diagnostics for resource-poor settings, could help with this (Figure 2).^7^ Mapping reliable laboratories and sharing microbiological data could also generate regional surveillance networks and local and regional antibiograms, which could refine empirical guidelines for sepsis, and inform antibiotic recommendations for non-urgent bacterial conditions.

### Pillar 3: antibiotic stewardship

MSF’s antibiotic stewardship programmes have trained clinicians to rationally use antibiotics in projects in the Middle East, from an orthopaedic reconstructive surgery project for conflict-related injuries (Amman, Jordan) to an acute trauma facility (Yemen), with future activities planned in Gaza and Iraq.^8^ On-site coaching is used to establish the programmes and ongoing, off-site clinical and operational support are always provided. However, human resources remain challenging in all of these settings. Point-of-care diagnostic tools would improve antibiotic targeting for inpatients and outpatients and could help clinicians manage expectations when antibiotics are requested but unnecessary. Policy changes must also improve prescription practices, address the widespread availability of over-the-counter broad-spectrum antibiotics and regulate markets that lack quality control. Governments must guarantee affordable antibiotics for patients that need them.

## Key elements for ABR project success

### Capacity building

Finding skilled human resources remains a primary challenge in addressing ABR in MSF settings, so building local capacity can create a degree of sustainability. MSF capacity-building endeavours are not passive learning experiences for staff but provide hands-on practical training and continued support. Often, a non-local IPC expert, (clinical) microbiologist or infectious disease specialist is sent to a field site for 3–6 months to identify and train a local team member to lead anti-ABR activities (IPC, microbiology and antibiotic stewardship). In the Middle East, ongoing ‘regional’ advisory positions provide clinical support to multiple countries’ projects and enhance the expertise being built in individual facilities. Coaching is provided by these regional experts about implementing IPC programmes, antibiotic activity spectrum, evidence-based antibiotic decision-making (based on infection site and severity), indications for microbiological testing and microbiology result interpretation.

MSF’s experience in Aden, Yemen, an acute trauma facility, demonstrates how feasible this approach can be. A Yemeni general practitioner was initially sent to MSF’s Jordan project to become familiar with an established, structured antibiotic stewardship programme.^3^ Upon return to Aden, on-site and remote coaching was provided by infectious disease and other specialists from both Amman and MSF headquarters. MSF-Aden now has a functioning antibiotic stewardship programme supporting its intensive care and surgical wards, providing daily feedback about laboratory results, and regular audits of prescribing practices (including surgical prophylaxis and treatment) and antibiotic consumption. A standardized training curriculum and e-learning tools are currently being developed, and MSF pathogen-specific antibiotic treatment protocols are being updated.

### Microbiology and access to care

Microbiological sampling, processing, and interpretation remains a cornerstone of antibiotic stewardship in MSF settings. Coaching is provided to local laboratory staff, and simplified, context-adapted laboratory standard operating procedures (SOPs) and quality assurance practices have been developed. Surgeons use SOPs for sampling intra-operative specimens for osteomyelitis diagnoses. Antibiotic stewards guide relevant clinicians using microbiological results, employing a collaborative stewardship approach that optimizes antibiotic prescription. Results are collated and reviewed to generate local antibiograms and inform empirical choices for sepsis treatment.

**Figure 3.**
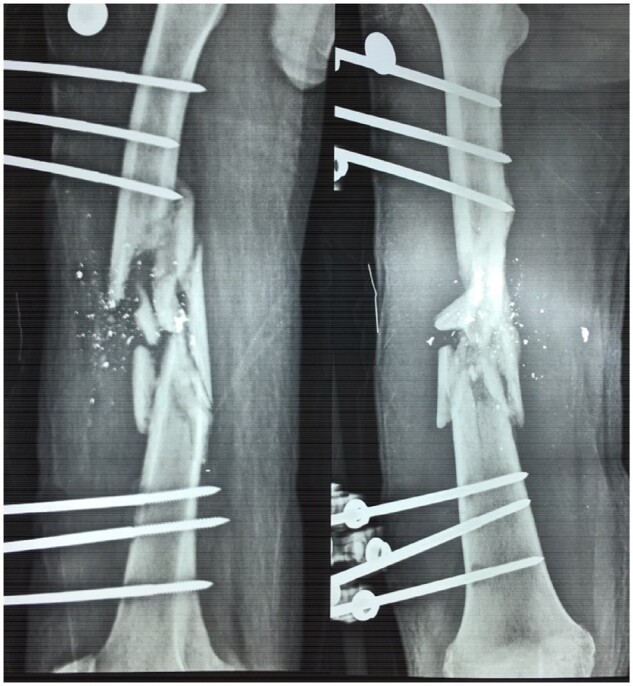
Compound fracture lower limb following gunshot injury; Gaza strip. © Rupa Kanapathipillai/MSF.

Maintaining high-quality microbiology in MSF contexts is not easy, however, and often demands flexibility. For example, in Gaza over 3000 patients with live ammunition gunshot wounds (of which 1400 are lower limb open fractures; Figure 3) receive care from MSF, and faster diagnosis and management of osteomyelitis patients is needed to keep up with the enormous caseload. Lacking sufficient microbiological capacity inside Gaza, MSF transports clinical samples across the border for processing in Tel-Aviv (Israel) weekly until this capacity is better established inside the Gaza Strip. In this context, it would be preferable for some patients to access care outside of Gaza for complex patient management (although the denial rate for patient exit permits remains significant), especially given the huge injury burden and multidisciplinary needs for reconstructive orthopaedic surgery, pain management, mental health, physiotherapy and nursing care. In Gaza, an adapted model of antibiotic stewardship is also needed to integrate inpatient and outpatient treatment, and outpatient parenteral antibiotic therapy (OPAT) is planned to counter the shortage of beds for osteomyelitis management.

## ABR in conflict and low-resource settings: work to be done

Ultimately, affordable access to existing antibiotics is essential to optimally treat patients in high-MDR settings, and cost remains an enormous burden (especially for carbapenems and fourth-generation cephalosporins) in MSF’s budget-constrained environments. Newer antibiotics and modes of delivery (such as portable infusers) hold promise and are being evaluated to find savings.

In projects where MSF has integrated ABR control measures, concrete action has been possible despite resource constraints and instability. But to prevent new resistant pathogens from emerging and to halt the spread of existing resistance, patient management must be optimized and addressed by all actors, including Ministries of Health in resource-poor and conflict settings. Field support from trained specialists, including via telemedicine, remains a key point of interaction that should be expanded, as should context-adapted, standardized ABR training and capacity-building tools for local staff. Shared protocols for prophylaxis and treatment in these contexts will help harmonize ABR approaches and introduce antibiotic stewardship. Ensuring affordable antibiotics and diagnostics supplies, sharing surveillance data and limiting over-the-counter availability of broad-spectrum antibiotics are all necessary steps to address ABR.

## Transparency declarations

None to declare.
